# Maintenance of Mitochondrial Morphology by Autophagy and Its Role in High Glucose Effects on Chronological Lifespan of *Saccharomyces cerevisiae*

**DOI:** 10.1155/2013/636287

**Published:** 2013-07-11

**Authors:** May T. Aung-Htut, Yuen T. Lam, Yu-Leng Lim, Mark Rinnerthaler, Cristy L. Gelling, Hongyuan Yang, Michael Breitenbach, Ian W. Dawes

**Affiliations:** ^1^School of Biotechnology and Biomolecular Sciences, University of New South Wales, Sydney, NSW 2052, Australia; ^2^Department of Genetics, University of Salzburg, Hellbrunnerstrare 34, 5020 Salzburg, Austria

## Abstract

In *Saccharomyces cerevisiae*, mitochondrial morphology changes when cells are shifted between nonfermentative and fermentative carbon sources. Here, we show that cells of *S. cerevisiae* grown in different glucose concentrations display different mitochondrial morphologies. The morphology of mitochondria in the cells growing in 0.5% glucose was similar to that of mitochondria in respiring cells. However, the mitochondria of cells growing in higher glucose concentrations (2% and 4%) became fragmented after growth in these media, due to the production of acetic acid; however, the fragmentation was not due to intracellular acidification. From a screen of mutants involved in sensing and utilizing nutrients, cells lacking *TOR1* had reduced mitochondrial fragmentation, and autophagy was found to be essential for this reduction. Mitochondrial fragmentation in cells grown in high glucose was reversible by transferring them into conditioned medium from a culture grown on 0.5% glucose. Similarly, the chronological lifespan of cells grown in high glucose medium was reduced, and this phenotype could be reversed when cells were transferred to low glucose conditioned medium. These data indicate that chronological lifespan seems correlated with mitochondrial morphology of yeast cells and that both phenotypes can be influenced by factors from conditioned medium of cultures grown in low glucose medium.

## 1. Introduction

Mitochondria are important organelles whose primary function is to synthesize ATP, but they also play important roles in many cellular processes including apoptosis and aging [1–4]. Due to their dynamic nature, the number and shape of mitochondria in a cell are variable depending on the growth conditions of the cell [5–7]. 

In *Saccharomyces cerevisiae*, the morphology of mitochondria is under the influence of the availability of oxygen and the nature of the carbon source for growth. Under anaerobic conditions, very small mitochondria known as promitochondria are observed. These are devoid of respiratory pigments and import ATP to perform the remaining essential metabolic functions [8]. On the other hand, enlarged tubular structures are found in aerobically grown cells [9]. *S. cerevisiae* cells respire in the absence of glucose, and these cells have a similar mitochondrial morphology to those observed in stationary phase cells where many small, round mitochondria are the dominant form [6, 10]. High glucose concentrations promote calcium and mitogen protein kinase-mediated activation of mitochondrial fission and stimulate reactive oxygen species production [11].

Restriction of glucose intake extends the cellular lifespan in a manner similar to caloric restriction [12, 13]. Alternatively, inhibition of nutrient signaling pathways by deletion of the *TOR1* gene or addition of rapamycin to growth media also extends both replicative and chronological lifespan (CLS) in *S. cerevisiae* [14, 15]. One of the downstream processes under regulation by the TOR pathway is autophagy, which is activated upon starvation or inhibition of TOR signaling [16]. Autophagy is conserved in all eukaryotic cells [17, 18] and it is important during starvation because it not only removes damaged organelles, but it also provides nutrients by recycling cellular constituents [19–21]. There is also increasing evidence that autophagy may play a role in lifespan extension in *Caenorhabditis elegans*, *Drosophila melanogaster, *and *S. cerevisiae* [22–24], especially during caloric restriction [25]. 

In neonatal rat ventricular myocytes, a high glucose concentration induced cell death via mitochondrial fragmentation possibly due to increased production of reactive oxygen species (ROS) [1]. Despite this interest in ageing, nutrients, and mitochondrial morphology, it remains to be determined whether there is any correlation between mitochondrial morphology and chronological ageing in *S. cerevisiae *over a wide range of glucose concentrations. We, therefore, investigated the mitochondrial morphology of yeast cells grown in different concentrations of glucose and sought to identify functions that are important in maintaining mitochondrial structure at elevated levels of glucose. 

## 2. Materials and Methods

### 2.1. Yeast Strains, Media, and Growth Conditions

All *S*. *cerevisiae* strains used were derived from BY4743 (*MATa/MAT*α* his3Δ1/his3Δ1 leu2Δ0/leu2Δ0 met15Δ0/MET15 LYS2/lys2Δ0 ura3Δ0/ura3Δ0*). Yeast strains were grown aerobically at 30°C in YPD (1% yeast extract, 2% peptone, and 2% glucose) or in synthetic complete medium SC (0.17% Difco yeast nitrogen base without amino acids and ammonium sulfate, 0.5% ammonium sulfate and 0.79 g/L amino acids mixture) supplemented with the indicated concentration of carbon source. The concentrations of amino acids used were according to [26]. For visualization of mitochondria, strains were transformed with the plasmid pUC35-*ACO1-*GFP and pUC35-*CIT1-Dsred* (gift from Professor Trevor. Lithgow, Monash University, Melbourne, VIC, Australia). Yeast strains harboring the plasmid pUC35-*ACO1-*GFP were grown in SC medium lacking uracil. For antibiotic selection, nourseothricin (ClonNAT, Werner BioAgents) or hygromycin B (Sigma-Aldrich) were added to a final concentration of 100 mg/L and 300 mg/L, respectively. Starter cultures were prepared by inoculating a single colony into 1.5 mL SC medium and incubating overnight at 30°C. The starter culture was then diluted to OD_600_ 0.1–0.15 in 2 mL fresh SC medium in a 10 mL tube for microscopic examination or 10 mL in a 50 mL tube for CLS and incubated at 30°C with shaking throughout the experiment. At the indicated intervals, 20 *μ*L culture was removed for microscopic examination and 100 *μ*L for serial dilution and spotting on a YPD plate. Five *μ*L of undiluted culture and of each dilution was spotted onto a YPD plate and incubated for 2-3 d at 30°C. Conditioned medium was prepared by growing the cells in SC medium containing different concentrations of glucose for 48 h and collecting the supernatant by centrifuging at 1800 ×g for 5 min. For media exchange experiments, the cells were grown for 6 h before pelleting and resuspending in the indicated conditioned media unless otherwise stated. The morphology of mitochondria was observed 2 h after media exchange. 

### 2.2. Measurement of Oxygen and Glucose Consumption

The rate of oxygen consumption was monitored using a standard 3 mL Clark-type oxygen electrode. The system was connected to a PowerLab data acquisition and analysis system (ADInstruments). Culture (2 mL) at the indicated time points was transferred to the chamber maintained at 30°C with constant stirring, and oxygen content was monitored for at least 10 min. For glucose consumption, the concentration of glucose in the supernatant was measured at 6, 24, 48, and 72 h after inoculation using an automated glucose analyzer (YSI 2300 STAT Plus Glucose & Lactate Analyzer). 

### 2.3. Microscopy

Mitochondrial morphology was observed using an Olympus BX60 fluorescence microscope at 100× magnification. An aliquot 5–10 *μ*L of sample was taken at indicated times, and micrographs of the cells with representative morphology were taken at room temperature. The images were acquired using IP lab software, and Adobe Photoshop was used to adjust the image size and the brightness and contrast. To determine the percentage of cells with total mitochondrial fragmentation within a population, the cells were grown for three days. An aliquot of sample was examined at indicated time points. The percentage of cells showing no visible tubular mitochondrial structure was determined by direct microscopic examination. For each assay at least 350 cells were counted, and the data are the mean of three independent experiments.

### 2.4. Gas Chromatography-Mass Spectrometry (GC-MS) Analysis

The GC-MS analysis was carried out using the Thermo Scientific DSQ II Single Quadrupole GC/MS at the Bioanalytical Mass Spectrometry Facility (BMSF), University of New South Wales. The samples were analyzed by GC-MS with the split injection mode and split ratio of 1 : 10. Carrier gas was helium at a constant flow rate of 1.5 mL/min. The inlet temperature was maintained at 240°C. GC oven temperature was held at 70°C for 1 min and then ramped to 140°C at 15°C/min where it was held for a further min. Mass spectrometry was carried out in positive ion mode using electron ionization and the mass spectra recorded within 41–74 amu.

### 2.5. Measurement of Intracellular pH

Intracellular pH was determined by the method of Brett et al. [27]. Strain BY4743 was transformed with pCB901YpHc containing the pHluorin gene (gift from Professor Rajini Rao, Johns Hopkins University School of Medicine, Baltimore) and pUG35 (non-pH sensitive GFP). The cells were grown in different glucose concentrations for 24 hours, and the intracellular pH was analyzed using a FACSCanto II (BD Biosciences). The fluorescent signals were collected at two different channels: Alexa Fluor 488 (488 nm) and AmCyan (405 nm). A calibration curve of the ratio of fluorescent intensities of 405/488 nm versus pH was obtained as follows. Sample (50 *μ*L) was diluted in 1 mL of medium containing 50 mM MES, 50 mM HEPES, 50 mM KCl, 50 mM NaCl, 0.2 M ammonium acetate, 10 mM NaN_3_, 10 mM 2-deoxyglucose, 75 *μ*M monensin, and 10 *μ*M nigericin, titrated to eight different pH values within the range of 5.0–8.0. The background was subtracted using the cells with pUG35, and the value of 405/488 was calculated using FlowJo software for each individual pH. The intracellular pH of the cells growing in different concentrations of glucose was estimated by comparing the ratio of fluorescent intensities of 405/488 nm obtained for the cells with the calibration curve. 

### 2.6. Measurement of Intracellular Acetate

The intracellular acetate concentration was measured for cells of the wild type and the Δ*atg1* mutant grown in different concentrations of glucose for 24 hours using the commercial kit from R-Biopharm (Cat. no. 10148261035) according to the manufacturer's instructions. 

### 2.7. Dihydroethidium (DHE) Staining and FACS Analysis

Staining with DHE (Molecular Probes) was performed as described by [28]. Briefly, cells (500 *μ*L) were stained with 5 *μ*g/mL final concentration of DHE for 10 min and analyzed using a FACSort (BD Bioscience). Analysis was performed for 20,000–30,000 cells per sample. All analyses were performed twice independently.

## 3. Results

### 3.1. Mitochondrial Morphology Changes in Response to Different Glucose Concentrations

In order to determine whether increasing glucose concentration also has an effect on mitochondrial structure and how this correlates with cellular lifespan in *S. cerevisiae*, we monitored the changes in mitochondrial morphology in *S. cerevisiae* cells growing in calorie-restricted (0.5%) and high glucose conditions (2% and 4%). *S. cerevisiae* cells were transformed with an aconitase-GFP fusion construct, *ACO1-*GFP, and expression of GFP was used to visualize mitochondrial structure. The use of this construct has been verified in [29].

**Figure 1:**
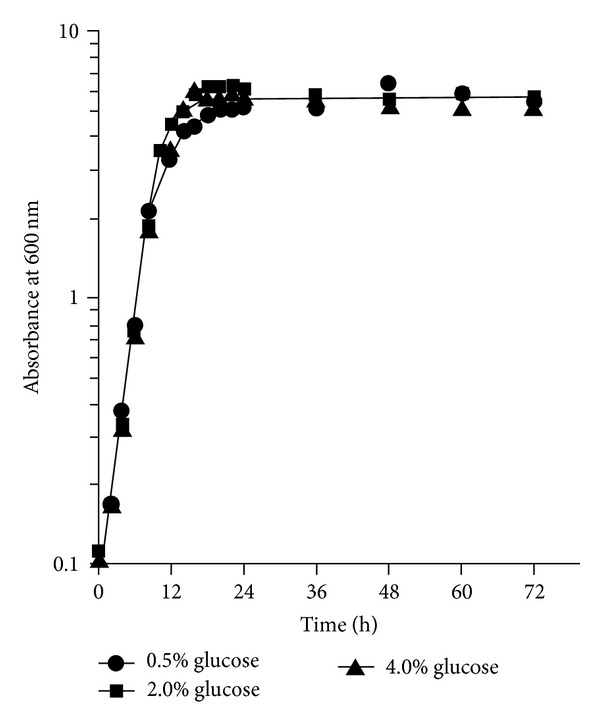
Growth of BY4743 overexpressing *ACO1-*GFP plasmid in SC medium supplemented with different concentrations of glucose. An overnight culture of cells in SC medium was diluted to an initial absorbance at 600 nm of 0.1 in fresh SC medium containing the glucose concentration indicated and the cultures incubated at 30°C with shaking. The cell concentration was estimated at intervals. Data are from a single experiment.

To ensure that mitochondrial morphology was examined at a similar growth phase, the growth of wild-type cells expressing *ACO1-*GFP in the different levels of glucose was monitored (Figure 1). A similar growth rate was observed for all glucose conditions and cells reached stationary phase at a comparable time. The final yields of these three levels of glucose culture were also similar.

**Figure 2:**
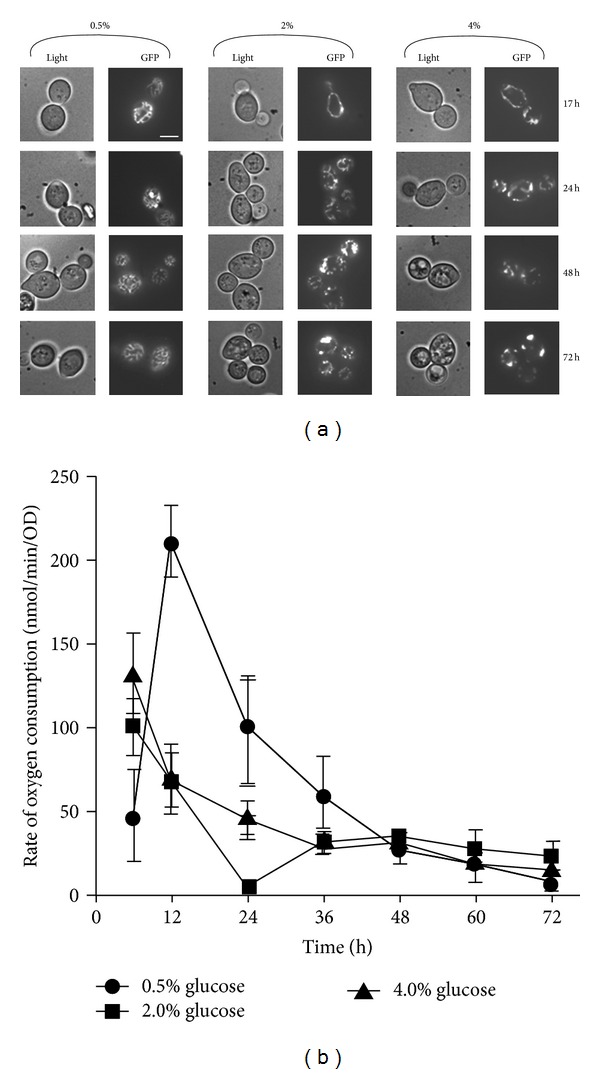
Mitochondrial morphologies of *S. cerevisiae* grown in different concentrations of glucose. (a) BY4743 wild-type cells transformed with an *ACO1*-GFP fusion construct were grown for 72 hours in three different concentrations of glucose (0.5%, 2%, and 4%), and the morphology of mitochondria was observed at the indicated times using a fluorescent microscope. The micrographs shown are representative of the population. Scale bar: 5 *μ*m. (b) The rates of oxygen consumption of these cells were also measured at the initial 6 hours after inoculation and at 12 hours intervals throughout the 72 hours time course. Data are the mean of three separate cultures in parallel. Error bars indicate the standard deviation.

Having determined the growth states of the cultures in the three glucose conditions, changes of mitochondrial morphology were examined. Cells were grown in synthetic medium (SC) containing 0.5%, 2%, or 4% glucose, and after 17 h growth, as cells entered the diauxic shift, mitochondrial morphology was examined using fluorescence microscopy. Remarkable differences in mitochondrial morphology were observed in response to changing glucose concentrations (Figure 2(a)). Under the standard laboratory condition with 2% glucose as a carbon source, mitochondria appeared as elongated tubular structures. However, in media containing 0.5% glucose, mitochondria displayed a highly branched, short-rod morphology similar to that observed in cells growing by respiration in ethanol medium [3]. In the highest level of glucose tested (4%), mitochondria displayed a partial bead-thread structure with very few connections and branches. Observations using a *CIT1-*DsRed construct instead of the *ACO1-*GFP construct also produced the same result, indicating that the effect of glucose concentrations on mitochondrial morphology was independent of the use of the aconitase-GFP fusion (data not shown).

The difference in mitochondrial morphologies between cells grown in 2% glucose and 4% glucose was independent of osmotic stress, since addition of an equivalent molar concentration of sorbitol to 2% glucose medium did not affect the mitochondrial appearance (data not shown). 

Having observed the characteristic mitochondrial morphology associated with glucose levels, we monitored the change of mitochondrial morphology in cells grown in 0.5%, 2% or 4% glucose media for 24, 48 and 72 hours (Figure 2(a)). Furthermore, to assess the structural changes of mitochondria, the percentage of cells in the population displaying total mitochondrial fragmentation, in which only punctate mitochondria with complete absence of tubular mitochondria within an individual cell, was determined (Table 1).

Cells grown in 2% or 4% glucose displayed an increased heterogeneity in mitochondrial morphology with time, showing a progression towards punctate fragmented structures over 72 hours (Figure 2(a)). After 24 hours of growth, the culture grown in 4% glucose had the highest number of cells with totally fragmented mitochondria (10%) followed by those grown in 2% glucose (7%) and 0.5% glucose (2%) (Table 1). The percentages of cells with totally fragmented mitochondria grown in 4% and 2% glucose increased to 64% and 66%, respectively, after 72 hours. However, cells under caloric restriction showed an average of less than 3% of the population with total mitochondrial fragmentation at that time.

We monitored respiratory rate under the above conditions to determine whether this affected the morphology of mitochondria. Maximal respiratory activity was observed in cells after 12 hours of growth in 0.5% glucose medium (Figure 2(b)). This respiration peak coincided with the presence of highly branched mitochondrial morphology observed in 0.5% glucose-grown cells. However, respiratory activity in these cells decreased from 24 hours to a low level at 72 hours, yet the highly branched mitochondrial morphology was maintained throughout the 72 hours time course. Therefore, a high rate of respiration was not required to maintain the highly branched mitochondrial morphology in these cells. On the other hand, the respiratory activity of 4% glucose-grown cells was relatively low and underwent a gradual decrease throughout the 72 h incubation. Since total mitochondrial fragmentation was observed in 4% glucose-grown cells as early as 24 hours of growth, decreasing the respiratory activity could not be the cause of the onset of mitochondrial fragmentation in the presence of high glucose levels.

**Figure 3:**
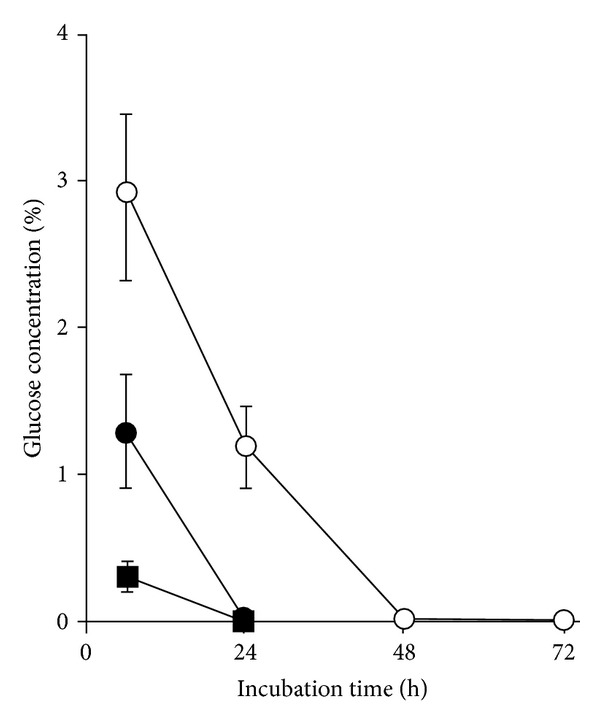
Glucose consumption of *S. cerevisiae* growing in 0.5%, 2%, and 4% glucose. Glucose in the culture medium was measured at intervals after incubation of the cells in SC medium containing glucose at 0.5% (closed squares), 2% (closed circles), or 4% (open circles). The measurements were performed on three separate cultures grown in parallel, and error bars indicate the SEM.

Glucose concentrations were measured in the supernatant collected from the different media at intervals throughout growth. The level of glucose was close to zero after 24 hours of growth in medium originally supplemented with 0.5% and 2% glucose, while cells consumed approximately half of the glucose in 4% glucose medium (Figure 3). These data indicate that there was no correlation between the concentration of glucose remaining in the medium and the progression of mitochondrial fragmentation. 

Together, the above data showed that neither growth state nor respiratory rate, and the rate of glucose consumption correlated with mitochondrial fragmentation observed in high glucose concentrations. We then further investigate the cause of early mitochondrial fragmentation in cells grown at high glucose concentrations (2% and 4%) by analyzing the mitochondrial morphology of cells lacking genes involved in maintaining mitochondrial morphology. 

### 3.2. Progression of Mitochondrial Fragmentation in High Glucose Is Independent of Mitochondrial Fission

Mitochondrial morphology is modulated by a delicate balance between mitochondrial fission and fusion, and in *S. cerevisiae,* deletion of the *DNM1 *gene involved in mitochondrial fission increases replicative lifespan [4]. We therefore determined whether the mitochondrial fragmentation observed in cells grown in a high level of glucose was regulated by factors affecting mitochondrial fission by examining mutant strains (*dnm1*Δ and *fis1*Δ) with known defects in the fission process. The *fis1*Δ strain used in these experiments has been shown to also carry a mutation in the *WHI2* gene which rescues the mitochondrial respiratory defect caused by FIS1 deficiency, which also causes a failure to suppress cell growth during amino acid deprivation [30]. The mutant cells were transformed with the *ACO1-*GFP construct and grown in 2% or 4% glucose medium under the same condition described above. 

The *dnm1*Δ mutant defective in mitochondrial fission was expected to show a reduced level of mitochondrial fragmentation [4]; however, when grown in 4% glucose, it displayed fragmentation comparable to that of the wild type (Table 1, see also Supplementary Figure S1 available online at http://dx.doi.org/10.1155/2013/636287). A slight reduction in the percentage of *dnm1*Δ cells that harbored fragmented mitochondria was observed when cells were grown in 2% glucose. Nevertheless, mitochondrial fragmentation progressed in the *dnm1*Δ strain under the high glucose conditions. Cells lacking *FIS1* also showed a reduction in the percentage of cells containing mitochondrial fragmentation when grown in 2% glucose. However, similarly to *dnm1*Δ, mitochondrial fragmentation was observed when *fis1*Δ cells were grown in 4% glucose, resulting in 63% of cells containing completely fragmented mitochondria. These results indicated that mitochondrial fragmentation was unavoidable when cells were grown in 4% glucose, even in cells defective in mitochondrial fission. Hence, mitochondrial fragmentation observed in high glucose levels was independent of mitochondrial fission. 

Cells deleted for the mitochondrial fusion gene *FZO1* lack mitochondrial DNA and had severely deformed mitochondria in both glucose concentrations examined, and it was therefore difficult to determine whether there was any involvement of mitochondrial fusion in the fragmentation of mitochondria using this mutant.

### 3.3. Inhibition of TOR Signaling Pathway Reduces Mitochondrial Fragmentation

Since nutrient availability might play a greater role than mitochondrial fission processes in modulating mitochondrial fragmentation when cells were grown at high glucose concentration, we examined mutant strains lacking genes involved in glucose sensing (*SNF3*, *RGT2*), glucose metabolism (*HXK2, GPA2*, *PDE1*, and *PDE2*), and general nutrient sensing (*TOR1*). Mutant cells transformed with the *ACO1-*GFP construct were grown in 2% or 4% glucose medium as described above.

Mutants with a deletion affecting glucose sensing or glucose metabolism showed 50% to 84% of cells with totally fragmented mitochondria morphology after 72 hours of growth in either 2% or 4% glucose (Table 1). Among the mutants screened, only cells lacking the *TOR1* gene showed a substantial reduction in the percentage of cells with totally fragmented mitochondria when grown in 2% or 4% glucose (Table 1; Supplementary Figure S2). Mitochondrial fragmentation of *tor1*Δ mutant cells was 42% and 37% when cells were grown in 4% glucose and 2% glucose, respectively, after 72 hours. As an alternative approach to genetic manipulation of the TOR pathway, the wild-type cells were treated with 10 nM rapamycin to inhibit both *TOR1 *and *TOR2 *gene products. Cells treated with rapamycin showed an even greater reduction in total mitochondrial fragmentation than in the *tor1*Δ strain, with only 12% to 15% of the cells showing totally fragmented mitochondria when the cells were grown for 72 hours in 2% or 4% glucose media containing 10 nM rapamycin, respectively (Table 1; Supplementary Figure S3). Hence, deletion of *TOR1 *only partially suppressed mitochondrial fragmentation while inhibition of the TOR pathway by rapamycin, which also inhibits *TOR2, *further repressed the extent of mitochondrial fragmentation during cell growth in high glucose levels.

### 3.4. Autophagy Is Required to Resist Mitochondrial Fragmentation Caused by Volatile Glucose Metabolites

Since mitochondrial fragmentation occurred after cells had grown in media, we tested whether cells grown in different concentrations of glucose excreted metabolites are capable of stimulating mitochondrial fragmentation. In order to test this hypothesis, conditioned medium (in which cells had been grown in 0.5% or 4% glucose for either 24 hours or 48 hours) was collected and then used to replace the growth medium of cells grown either to exponential (6 hours) or stationary phase (48 hours). 

**Figure 4:**
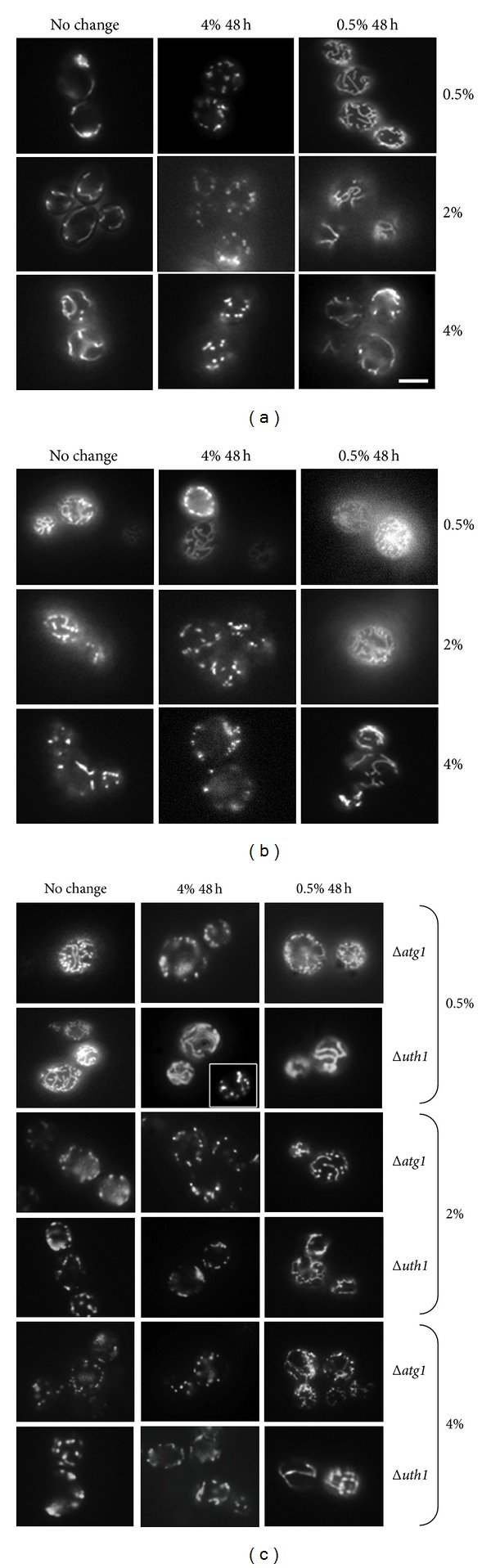
Conditioned medium from *S. cerevisiae* grown in 4% glucose triggered mitochondrial fragmentation, which was delayed by autophagy. (a) The wild-type cells pregrown to exponential phase for 6 hours in 0.5%, 2%, and 4% glucose to exponential phase were transferred to the 4% conditioned medium (4% 48 h) or 0.5% conditioned medium (0.5% 48 h), and mitochondrial morphology was observed. (b) The wild-type cells grown to stationary phase for 48 hours in 0.5%, 2%, and 4% glucose, then transferred to the 4% conditioned medium (4% 48 h) or 0.5% conditioned medium (0.5% 48 h), and mitochondrial morphology was observed. (c) Mutants affected in autophagy (Δ*atg1* and Δ*uth1*) were grown to stationary phase as under (b), and mitochondrial morphology was observed. Scale bar: 5 *μ*m. The micrographs shown are representative of the populations. Note: the micrograph for the Δ*uth1* mutant transferred into 4% 48 h represents the morphology of half the population of the cells, and the inset represents that of the other half of the population.

The conditioned medium that was initially supplemented with 4% glucose (4% glucose, 48 hours) contained substances that caused mitochondrial fragmentation in exponential phase cells, regardless of the glucose concentration of the medium in which the cells were pregrown (Figure 4). Mitochondrial fragmentation was observed as early as 2 h after transfer into this medium. Fragmentation also occurred for stationary phase cells pregrown in medium containing 2% and 4% glucose. In contrast, stationary phase cells pregrown in 0.5% glucose were resistant to mitochondrial fragmentation induced by the same medium. It was hypothesized that mitochondrial fragmentation was prevented in these cells because nutrients became depleted, and autophagy was activated earlier than in the other growth regimes.

In order to investigate the involvement of autophagy in resistance to conditioned medium-induced mitochondrial fragmentation, the autophagy mutant strains Δ*uth1*, Δ*atg1*, and Δ*atg5* were grown to stationary phase in medium containing 0.5% glucose and then transferred into the conditioned medium (4% glucose, 48 hours). The mitochondria of the Δ*atg1 *and Δ*atg5* mutants became fragmented, but not those of the mitophagy mutant Δ*uth1*. These results indicated that general autophagy was important for conferring resistance to the metabolites that stimulated mitochondrial fragmentation and that starvation may be able to delay mitochondrial fragmentation. Indeed, delayed fragmentation was observed in the cells growing in 10-fold diluted SC medium containing 2% glucose compared to the cells growing in normal SC medium with 2% glucose (data not shown).

**Figure 5:**
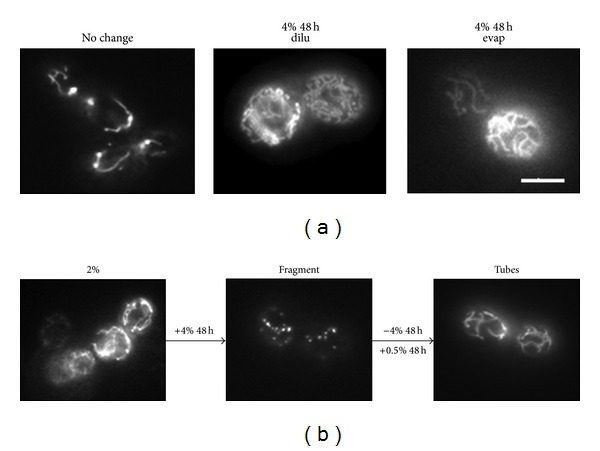
Reversibility of conditioned medium-induced mitochondrial fragmentation. (a) The mitochondria of the wild-type cells grown in 2% glucose did not fragment when they were transferred into diluted (4% 48 h dilu) and evaporated (4% 48 h evap) conditioned media (4% glucose, 48 hours). Mitochondria were observed at 2 hours, 20 hours, and 62 hours after media exchange. Only the micrographs taken at 20 hours after medium exchange are shown. (b) Mitochondrial fragmentation of the wild-type cells grown in 2% glucose was triggered by the 4% conditioned medium (+4% 48 h). Cells were grown in the 4% conditioned medium for 24 hours before transferring into the 0.5% conditioned medium (+0.5% 48 h). The morphology of mitochondria was observed 2 hours after transfer. Scale bar: 5 *μ*m. The micrographs shown are representative of the populations.

Since conditioned medium (4% glucose, 48 hours) appeared to contain a substance that stimulated fragmentation of mitochondria, it was analysed further. Treatment with diluted spent medium did not cause mitochondrial fragmentation in *S. cerevisiae* pregrown in any of the glucose concentrations (Figure 5(a)), indicating that the effect was probably not due to the physical disturbance of changing the medium but due to the concentration of the glucose metabolites present. These cells maintained tubular mitochondrial structure for at least 62 hours after the medium was exchanged. In addition, vacuum evaporation of the conditioned medium rendered it unable to stimulate mitochondrial fragmentation (Figure 5(a)) indicating that the stimulatory substance/s were volatile. Interestingly, mitochondrial fragmentation stimulated by the conditioned medium was found to be reversible once the medium was removed (Figure 5(b)).

### 3.5. The Observed Mitochondrial Fragmentation Was Not due to Intracellular Acidification

Since the metabolite(s) responsible for mitochondrial fragmentation was(were) volatile, we analysed all of the 48 hours conditioned media (0.5% glucose, 2% glucose, and 4% glucose) by gas chromatography-mass spectrometry. Three volatile substances with higher concentrations in the 4% glucose-conditioned medium were detected: acetic acid, ethanol, and 2,3-butanediol (Supplementary Table S1). Of the three compounds, acetic acid was the only one that resulted in mitochondrial fragmentation when separately added to the cells (Supplementary Figure S4). 

Mitochondrial fragmentation triggered by acetic acid treatment could be due to intracellular acidification caused by release of protons or to accumulation of acetate. In addition to acetic acid, benzoic acid and 2,4-dinitrophenol (2,4-DNP) also triggered mitochondrial fragmentation (Figure S1). One feature that is common to these three compounds is their ability to lead to acidification within the cells, and therefore we analyzed the intracellular concentration of acetate and intracellular pH of cells grown in different glucose concentrations. The intracellular level of acetate was higher in the wild-type cells growing in 2% and 4% glucose than those growing in 0.5% (See Supplementary Table S2). The intracellular pH of the cells grown in different concentrations of glucose was measured using the pH-sensitive GPF probe pHluorin. No significant correlation between intracellular pH and mitochondrial fragmentation was found (Supplementary Figure S5). Although mitochondrial fragmentation was already established in 2% and 4% glucose-grown cells within 24 hours of inoculation (Table 1), the intracellular pH of these cells was similar to that of 0.5% glucose-grown cells at that time. These results indicated that intracellular acidification was unlikely to be responsible for triggering mitochondrial fragmentation and that acetate or some metabolite derived from it is more likely to be responsible.

### 3.6. Autophagy Is Required to Reduce Mitochondrial Fragmentation

One of the many cellular processes regulated by the TOR pathway is autophagy, which recycles damaged proteins and organelles and makes amino acids and other essential metabolites to the cell [31] available. To determine whether autophagy plays a role in the reduction of mitochondrial fragmentation under high glucose conditions, a mutant strain defective for initiation of autophagy (*atg1*Δ) was transformed with *ACO1-*GFP construct to examine mitochondrial fragmentation (Table 1).

Cells deleted for *ATG1* displayed higher percentages (approximately 75% after 72 hours incubation) of mitochondrial fragmentation than the wild type, indicating that autophagy acts to reduce the onset of mitochondrial fragmentation in 2% and 4% glucose-grown cells. Since autophagy was important in maintaining mitochondrial morphology under these conditions, cells lacking genes affecting mitochondrial-specific autophagy, *ATG32 *[32, 33], and *UTH1* [34] that is also affected in cellular ageing [35], were analyzed. Surprisingly, deletion of *UTH1* or *ATG32 *did not affect mitochondrial fragmentation compared to that in the wild-type cells, indicating that mitochondrial-specific autophagy alone did not substantially suppress mitochondrial fragmentation. However, general autophagy, involving *ATG1* appears to play a vitally important role for reducing mitochondrial fragmentation under higher glucose conditions since deletion of *ATG1* elevated the fragmentation of mitochondria seen in cells grown on higher glucose levels. 

Subsequently, we checked whether the TOR pathway regulated the function of autophagy in reducing mitochondrial fragmentation. The autophagy mutants were treated with rapamycin, and total mitochondrial fragmentation was examined. A reduction of mitochondrial fragmentation in rapamycin-treated *uth1*Δ was observed (Table 1), which was consistent with the finding that deletion of *UTH1* did not have an impact on mitochondrial fragmentation and that the suppression effect of rapamycin observed was independent of *UTH1. *In the *atg1*Δ mutant, although treatment with rapamycin reduced mitochondrial fragmentation compared to the untreated mutant cells, the level of mitochondrial fragmentation remained much higher than in rapamycin-treated wild-type cells. Hence, rapamycin inhibition of the TOR pathway led to suppression of mitochondrial fragmentation, but this was largely dependent on the presence of a functional autophagy pathway. Therefore, it is likely that autophagy functions downstream of the TOR pathway in maintaining mitochondria in a nonfragmented state.

### 3.7. Role of Autophagy in Mitochondrial Fragmentation Induced by Glucose Metabolites

Having identified a cellular process that is able to prevent mitochondrial fragmentation in cells grown in high glucose concentrations, we next examined what triggered mitochondrial fragmentation in these cells. Mitochondria are the major site of reactive oxygen species (ROS) production, and an elevation of ROS could be one of the causes of mitochondrial fragmentation. We examined the levels of superoxide anion by DHE staining of cells growing in 0.5%, 2%, and 4% glucose over a 72 h time course and flow cytometry analysis to determine whether elevation in superoxide levels was correlated with the occurrence of mitochondrial fragmentation. 

**Figure 6:**
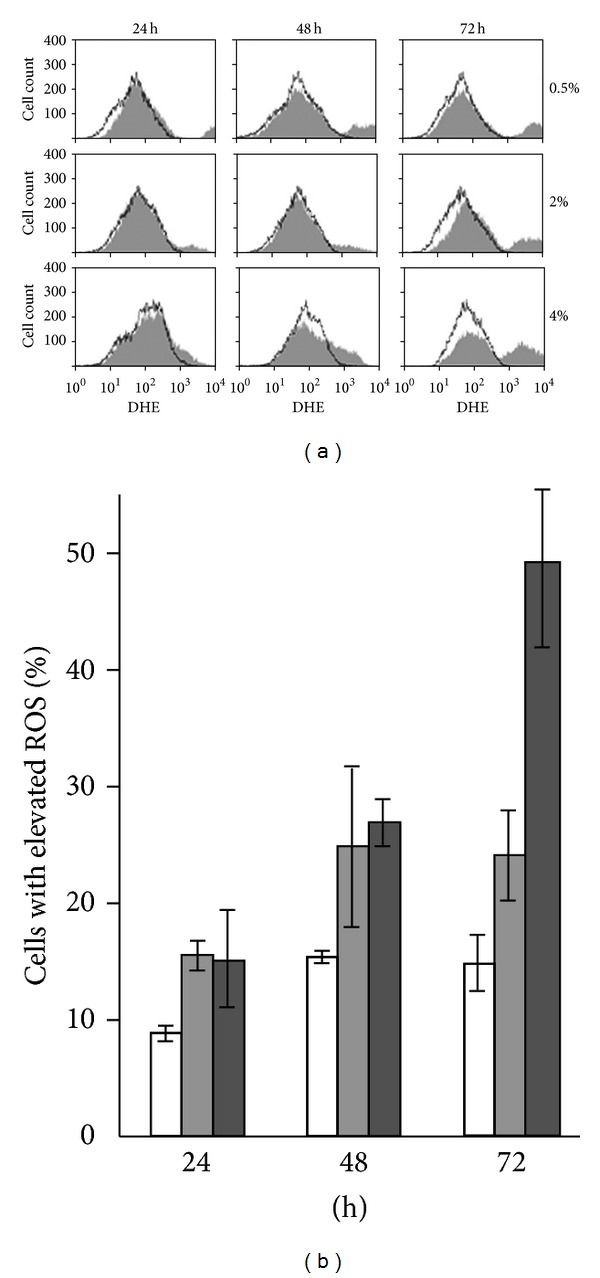
ROS levels in *S. cerevisiae* at 24 h, 48 h, and 72 h of growth. Wild-type cells grown in 0.5%, 2%, and 4% glucose were collected at the indicated times and stained with 5 *μ*g/mL DHE to detect superoxide radicals. Fluorescence intensities were analyzed by flow cytometry. (a) The clear and filled histograms represent the cells without and with DHE, respectively. (b) Percentage cells showing elevated ROS levels at each incubation time for cells grown in 0.5% glucose (clear rectangles), 2% glucose (lighter grey rectangles), and 4% glucose (darker grey rectangles). Data are the averages from two independent experiments; bars indicate the range of data obtained.

Cellular superoxide levels increased over time regardless of the concentration of glucose, as shown in Figure 6. Cells grown in 0.5% glucose had the highest superoxide level after 24 hours growth, which is consistent with the fact that respiratory activity was the highest for these cells at that time (Figure 2(b)). After 72 h incubation, cells grown in 4% glucose had the highest level of superoxide followed by those grown in 2% glucose and then those grown in 0.5% glucose. It is therefore unlikely that an increase in ROS level triggered mitochondrial fragmentation during cell growth, since the onset of elevated levels of ROS in 0.5% glucose-grown cells did not lead to mitochondrial fragmentation. 

Since fragmentation occurred 24 hours after inoculation in media, we analyzed whether the glucose metabolites accumulated in the medium during growth stimulated mitochondrial fragmentation. To test this hypothesis, conditioned medium (in which cells were grown for either 24 hours or 48 hours) originating from 4% glucose or 0.5% glucose medium was collected and then was used to replace the growth medium of cells grown to either exponential (6 hours) or stationary phase (48 hours). 

The 48 hours conditioned medium that was initially supplemented with 4% glucose (4% conditioned medium) contained substances that caused mitochondrial fragmentation in wild-type cells in exponential phase, regardless of the glucose concentration of the medium in which cells were pregrown (Figure 4(a)). For instance, mitochondrial fragmentation was observed as soon as two hours after transferring cells into the 4% conditioned medium. This fragmentation was also found for stationary phase wild-type cells pregrown in medium containing 2% or 4% glucose (Figure 4(b)). In contrast, the stationary phase cells pregrown in 0.5% glucose did not display fragmented mitochondria after transfer into the 4% conditioned medium (Figure 4(b)). We hypothesized that the early nutrient depletion in 0.5% glucose-grown cells activated autophagy leading to resistance to induction of mitochondrial fragmentation. 

In order to investigate the involvement of autophagy in this mitochondrial fragmentation process, the *atg1*Δ and *uth1*Δ autophagy mutants were grown for 48 hours in 0.5%, 2%, and 4% glucose and then transferred into the conditioned media (Figure 4(c)). Mitochondrial fragmentation was observed in the *atg1*Δ mutant cells, including those pregrown in 0.5% glucose, after transfer into the 4% glucose conditioned medium. On the other hand, *uth1*Δ cells pregrown in 0.5% glucose medium were partially resistant to 4% conditioned medium-induced mitochondrial fragmentation (approximately 50% of the total population displayed tubular mitochondria). These results indicated that activation of general autophagy during starvation played an important role in conferring resistance to those metabolites present in the conditioned medium that stimulated mitochondrial fragmentation. 

Conversely, wild-type cells transferred into 0.5% conditioned medium displayed tubular mitochondria independent of the growth phase and the level of glucose in which the cells were pregrown (Figure 4(c)). The fragmented mitochondria in the *atg1*Δ, and* uth1*Δ mutants also returned to a tubular structure after cells were transferred into 0.5% conditioned medium, although these cells required a longer time for recovery. 

The effects seen using 4% conditioned medium to stimulate fragmentation of mitochondria were not due to the physical disturbance of changing the medium. When conditioned medium was removed and fresh medium was supplemented to cells, there was no fragmentation in cells pregrown in any of the glucose concentrations used. Interestingly, mitochondrial fragmentation stimulated by the 4% conditioned medium was found to be reversible once the medium was replaced by the 0.5% conditioned medium (Figure 5). The reversible nature of the process indicated that the cells were not yet committed to any deleterious effects that may result from mitochondrial fragmentation.

### 3.8. Mitochondrial Fragmentation and Chronological Lifespan

The above results demonstrated that *S. cerevisiae* cells grown in high glucose concentrations not only possessed fragmented mitochondria but also showed higher levels of oxidative stress than those grown in calorie-restricted conditions. It is well known that *S. cerevisiae* cells that are restricted in their calorie intake have longer chronological and replicative lifespans [36, 37], that maintenance of the morphology of mitochondria is important for cell survival since the mutants that preserve tubular mitochondrial structure (such as *Δdnm1*) live longer than the wild-type cells [4], and the mutants that progress early to mitochondrial fragmentation have shorter lifespan [38]. This led us to investigate whether reversing fragmentation of mitochondria of cells grown in 2% and 4% glucose would extend their lifespan. 

Since mitochondrial fragmentation in 4% or 2% glucose-grown cells could be reversed in 0.5% conditioned medium (Figure 5(b)) and vice versa (Figure 5(a)), we determined whether chronological lifespan (CLS) could also be reversed in the same way. Cells were grown in 0.5%, 2%, or 4% glucose for 48 hours and then transferred into conditioned media as shown in Figure 6, and their CLS were assessed. 

**Figure 7:**
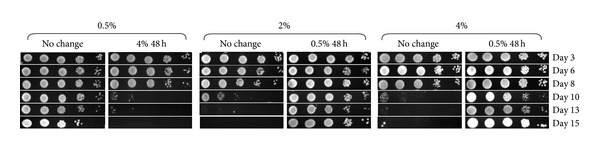
The shortened chronological lifespan of *S. cerevisiae *cells grown in higher concentrations of glucose can be reversed without genetic manipulation. Wild-type cells were grown in media containing 0.5%, 2%, and 4% glucose for 48 hours and exchanged into the conditioned media originally supplemented with 0.5% (0.5% 48 h) or 4% (4% 48 h) glucose. Cell viability was assessed by spotting diluted cultures onto YPD plates at indicated times and compared with that of the cells without any media exchange (no change).

As expected, cells grown in 0.5% glucose had an extended CLS compared to those grown in higher glucose concentrations (Figure 7). Interestingly, their lifespan was shortened when these cells were transferred into 4% conditioned medium. On the other hand, the lifespan of cells grown in 4% glucose medium was extended following their transfer into 0.5% conditioned medium. Burtner et al. [39] have also shown that the CLS was reversible by substituting spent growth medium in a similar way. Here, we show that the CLS of *S. cerevisiae *varied depending on the type of medium into which cells were exchanged and that this correlated with the reversible changes in mitochondrial morphology.

## 4. Discussion

Mitochondrial morphology is dynamic and responds to changes in a cell's physiology and metabolism. This morphology is modulated by the balance between fusion and fission processes [5, 40, 41]. However, factors such as apoptotic signals or oxidative stress cause an imbalance between these two processes resulting in fragmentation of mitochondria to a punctate morphology [6, 26, 42, 43], and this altered morphology occurs on induction of cell death and during ageing of cells [44]. Here, we have shown that mitochondrial morphology of *S. cerevisiae* changes depending on the concentration of glucose in the medium and that high glucose availability triggers mitochondrial fragmentation in yeast. This process is largely independent of mitochondrial fission or mitochondrial respiratory activity, instead reflecting nutrient sensing mechanisms involving the TOR pathway via the general autophagy process that it modulates. Early onset of respiratory activity correlated with an increased level of ROS in cells grown in low glucose levels as expected, but it is unlikely that mitochondrial damage caused by an increasing level of ROS is a trigger for mitochondrial fragmentation since cells grown in low glucose were respiring, producing relatively high levels of ROS (superoxide anion) yet maintained their mitochondrial structure during prolonged incubation. 

To investigate possible mechanisms involved in maintaining the mitochondrial structure, a set of deletion mutants was examined for the phenotype of reduced mitochondrial fragmentation in the presence of higher concentrations of glucose. The mutant lacking *TOR1 *was the only one examined that repressed mitochondrial fragmentation under higher glucose levels (2% and 4%). Wild-type cells treated with rapamycin also displayed nonfragmented mitochondria for a prolonged period under these conditions. Together, these results demonstrated that regulation of the TOR pathway plays a major role in the maintenance of mitochondrial structure. Both caloric restriction and inhibition of TOR delayed fragmentation in an autophagy-dependent manner since deletion of the *ATG1* gene led to an increased fragmentation under both conditions. Interestingly, selective elimination of damaged mitochondria by mitophagy in response to mitochondrial dysfunction [36] by mutating the *UTH1* or *ATG32 *genes did not affect the fragmentation of mitochondria observed in higher glucose-grown cells. Hence, the protective effect of autophagy is most likely by modulation of nutrient status, or elimination of cellular damage to components other than mitochondria, since they are degraded only by selective autophagy [45]. It is possible that the early low level of ROS observed in respiring cells in 0.5% glucose condition may have activated autophagy to maintain mitochondrial structure under this condition [46]. 

The importance of autophagy in cellular lifespan extension is highlighted by the demonstration of its importance in *C. elegans* during dietary restriction [25] and that autophagy and amino acid homeostasis are required for extended CLS in *S. cerevisiae* [24]. Autophagy is necessary for rapamycin-induced lifespan extension [47]. Induction of autophagy by spermidine also increases longevity in yeast, flies, and human cells [48]. Based on our data, the prevention or delay of the onset of mitochondrial fragmentation by autophagy may play an important role in lifespan extension. 

It is interesting that both the fragmentation of mitochondria and the shortening of CLS of cells grown in high glucose condition are reversible. Fragmented mitochondria in cells were able to return to a tubular morphology, and the cellular lifespan was extended when high glucose medium was replaced by the conditioned medium originating from cells grown on low glucose. The reversibility with respect to CLS has also been shown [39]. For mitochondrial morphology this change occurred within 2 hours of replacement. This reversibility of mitochondrial fragmentation is dependent on general autophagic processes. Further study on the composition of the conditioned media and comparison between the one that shortens lifespan and the one that lengthens may reveal which factor(s) present in the medium cause early cell death. These results further point to the correlation between mitochondrial morphology and chronological lifespan in *S. cerevisiae*, which has relevance to the effects of glucose and/or caloric restriction on cell aging. 

## Supplementary Material

The supplementary Tables S1 and S2 include data relative to: the volatile components produced by growth of cells to stationary phase in media containing different concentrations of glucose. The Supplementary Figures illustrate: the effects of mutations in mitochondrial fission/fusion on mitochondrial morphology (Fig. S1); the effect on mitochondrial morphology of mutating or inhibiting the TOR pathway (Figs. S1 and S2); the effects of compounds leading to intracellular acidification on mitochondrial morphology (Fig. S4); and, the intracellular pH of cells grown in media containing different concentrations of glucose (Fig S5).
Table S1 provides an estimation of the concentrations of the main volatile metabolites (ethanol, acetic acid and 2,3-butanediol in conditioned media from cells grown in SC medium containing 0.4%, 2% or 4% glucose.
Table S2 gives the intracellular acetate concentration in the wild-type and mutant cells grown for 72 h in SC medium containing different concentrations of glucose.
Figure S1 illustrates the mitochondrial morphology in cells of the wild type and *dnm1*, *fis1* and *fzo1* mutants defective in mitochondrial fission and fusion.
Figure S2 illustrates that deletion of TOR1 protects cells against mitochondrial fragmentation.
Figure S3 shows that rapamycin treatment also prevents mitochondrial fragmentation.
Figure S4 Acetic acid, benzoic acid and 2,4-DNP triggered mitochondrial fragmentation in *S. cerevisiae*.
Figure S5 gives the intracellular pH of cells grown in different concentrations of glucose.

## Figures and Tables

**Table 1:** The percentage of wild-type and mutant cells showing completely fragmented mitochondrial morphology in cultures incubated in three different concentrations of glucose (0.5%, 2%, and 4%) at the times indicated.

Strain	0.5% glucose			2% glucose			4% glucose		
	24 h	48 h	72 h	24 h	48 h	72 h	24 h	48 h	72 h
Wild type	1.6 (0.7)	2.4 (0.9)	2.2 (1.2)	6.5 (0.8)	36.0 (1.1)	63.6 (4.3)	10.7 (2.1)	38.2 (7.5)	66.2 (6.7)

Δ*dnm1 *	0.8 (1.3)	0.8 (0.9)	2.9 (1.2)	1.3 (1.5)	22.4 (1.0)	45.1 (1.3)	3.6 (1.5)	29.5 (2.2)	67.5 (1.9)
Δ*fis1 *	n.d.	n.d.	n.d.	1.8 (1.1)	7.3 (2.1)	30.2 (1.5)	6.3 (0.8)	13.3 (1.2)	63.2 (5.7)

Δ*tor1 *	1.0 (1.0)	0.9 (0.3)	1.5 (0.7)	6.1 (0.3)	8.2 (1.1)	36.7 (0.9)	11.3 (1.2)	27.0 (1.0)	41.4 (1.0)

Δ*gpa2 *	1.7 (0.6)	1.2 (0.4)	4.9 (0.6)	6.3 (0.6)	39.7 (3.7)	67.7 (1.7)	5.6 (1.6)	34.7 (1.0)	54.8 (1.0)
Δ*snf3 *	1.9 (0.4)	3.8 (0.5)	6.0 (0.7)	5.5 (0.7)	32.9 (3.1)	47.7 (2.3)	8.5 (1.9)	71.2 (4.2)	75.4 (5.4)
Δ*rgt2 *	n.d.	n.d.	n.d.	5.3 (1.9)	28.8 (3.6)	50.7 (2.6)	8.3 (2.2)	22.7 (2.9)	72.4 (6.2)
Δ*pde1 *	0.8 (0.7)	1.0 (0.4)	1.3 (1.2)	11.9 (1.5)	60.0 (2.0)	78.4 (1.6)	16.3 (3.1)	61.7 (2.2)	70.5 (2.9)
Δ*pde2 *	4.8 (0.1)	3.8 (0.9)	9.3 (4.3)	6.8 (1.5)	56.1 (5.4)	84.6 (1.7)	16.6 (4.8)	48.0 (8.4)	68.1 (2.9)

Δ*uth1 *	3.1 (1.7)	2.9 (0.4)	6.8 (3.0)	7.5 (2.2)	37.0 (4.4)	54.9 (2.6)	14.0 (3.3)	34.0 (7.5)	63.0 (5.1)
Δ*atg32 *	n.d.	n.d.	n.d.	8.2 (1.7)	27.6 (2.8)	50.1 (4.2)	9.3 (1.5)	60.2 (1.6)	68.4 (4.3)
Δ*atg1 *	14.8 (5.2)	8.0 (0.7)	12.0 (1.7)	14.4 (1.4)	57.7 (1.0)	74.6 (0.8)	13.0 (2.2)	60.5 (7.2)	74.4 (0.5)

Wild type + rapamycin^1^	2.8 (0.2)	3.2 (0.3)	4.2 (0.7)	3.9 (0.6)	6.2 (1.3)	12.0 (0.9)	5.6 (1.5)	7.6 (1.0)	14.9 (0.9)
Δ*uth1* + rapamycin^1^	2.5 (0.6)	2.6 (0.8)	2.1 (0.4)	2.5 (0.7)	3.6 (0.5)	5.9 (0.7)	3.8 (0.9)	6.1 (0.5)	17.2 (1.6)
Δ*atg1* + rapamycin^1^	7.4 (0.5)	10.1 (1.5)	13.5 (1.6)	9.0 (0.4)	30.8 (1.4)	62.5 (0.9)	12.9 (0.8)	31.1 (1.8)	67.4 (0.7)

^1^Wild-type cells or mutants were grown in the presence of 10 nM rapamycin.

*The *fis1*Δ strain used in these experiments has been shown to also carry a mutation in the *WHI2* gene which rescues the mitochondrial respiratory defect caused by *FIS1* deficiency, but which also causes a failure to suppress cell growth during amino acid deprivation [29].
