# Supplementary material for: Maintenance of Mitochondrial Morphology by Autophagy and Its Role in High Glucose Effects on Chronological Lifespan of Saccharomyces cerevisiae
Source: Oxid Med Cell Longev. 2013 Jul 11;2013:636287. doi: 10.1155/2013/636287 (PMC3727090; doi:10.1155/2013/636287)
Supplement: Supplementary file 1 — The supplementary Tables S1 and S2 include data relative to: the volatile components produced by growth of cells to stationary phase in media containing different concentrations of glucose. The Supplementary Figures illustrate: the effects of mutations in mitochondrial fission/fusion on mitochondrial morphology (Fig. S1); the effect on mitochondrial morphology of mutating or inhibiting the TOR pathway (Figs. S1 and S2); the effects of compounds leading to intracellular acidification on mitochondrial morphology (Fig. S4); and, the intracellular pH of cells grown in media containing different concentrations of glucose (Fig S5). Table S1 provides an estimation of the concentrations of the main volatile metabolites (ethanol, acetic acid and 2,3-butanediol in conditioned media from cells grown in SC medium containing 0.4%, 2% or 4% glucose. Table S2 gives the intracellular acetate concentration in the wild-type and mutant cells grown for 72 h in SC medium containing different concentrations of glucose. Figure S1 illustrates the mitochondrial morphology in cells of the wild type and dnm1, fis1 and fzo1 mutants defective in mitochondrial fission and fusion. Figure S2 illustrates that deletion of TOR1 protects cells against mitochondrial fragmentation. Figure S3 shows that rapamycin treatment also prevents mitochondrial fragmentation. Figure S4 Acetic acid, benzoic acid and 2,4-DNP triggered mitochondrial fragmentation in S. cerevisiae. Figure S5 gives the intracellular pH of cells grown in different concentrations of glucose. [file 636287.f1.doc]

**Supplementary Tables**

**Table S1.** **Selected volatile metabolites detected in conditioned media using gas chromatography mass spectrometry.** Cells of the wild type were grown for 48 h in SC medium containing different concentrations of glucose.

| Samples | Ethanol | Acetic acid | 2,3- butanediol |
| --- | --- | --- | --- |
| 0.5% 48 h | 0.053 (0.04) | 0.089 (0.046) | ND |
| 2% 48 h | 1 (0.23) | 1 (0.37) | 1 (0.52) |
| 4% 48 h | 3.98 (2.15) | 3.76 (1.12) | 6.87 (1.02) |

ND = Not detectable. The mean values and s.d. (in brackets) are from data from three independent experiments. The values are shown as fold difference compared to the level of each metabolite detected in the conditioned medium originally containing 2% glucose (2% 48 h). 0.5% glucose conditioned media; 0.5% 48 h, 4% glucose conditioned media; 4% 48 h.

**Table S2** **Intracellular acetate concentration in the wild type and mutant cells grown for 72 h in SC medium containing different concentrations of glucose.**

| Strain | 0.5% glucose | 2% glucose | 4% glucose |
| --- | --- | --- | --- |
| Wild-type | 0.08 | 0.30 | 0.23 |
| *Δatg1* | 0.16 | 0.26 | 0.34 |

Acetate concentration is expressed as g/100g of cells. The values are averages from two independent experiments.

**Supplementary Figures**

**
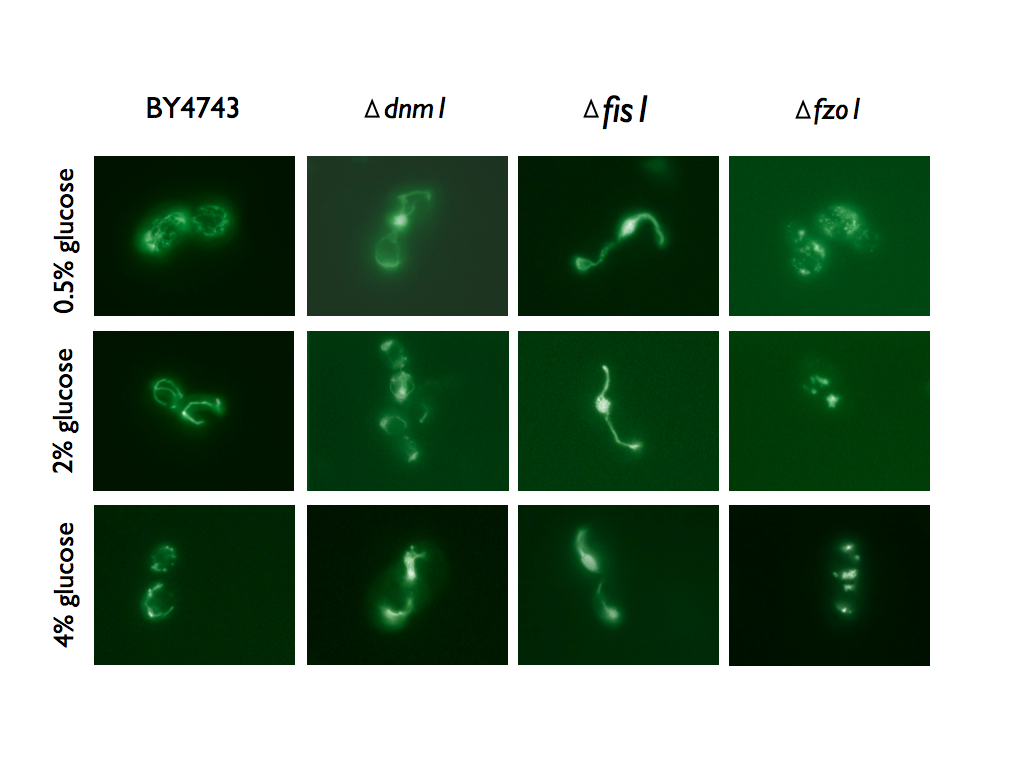
**

**Figure S1. Mitochondrial morphology in cells of the wild type and mutants defective in mitochondrial fission and fusion.** Wild-type, *Δdnm1*,  *Δfis1* and *Δfzo1* mutant cells transformed with the *ACO1*-GFP fusion construct were grown for 72 hours in three different concentrations of glucose (0.5%, 2%, and 4%) and the morphology of mitochondria was observed using a fluorescent microscope. The micrographs illustrate the fragmentation as discussed in the text.

**
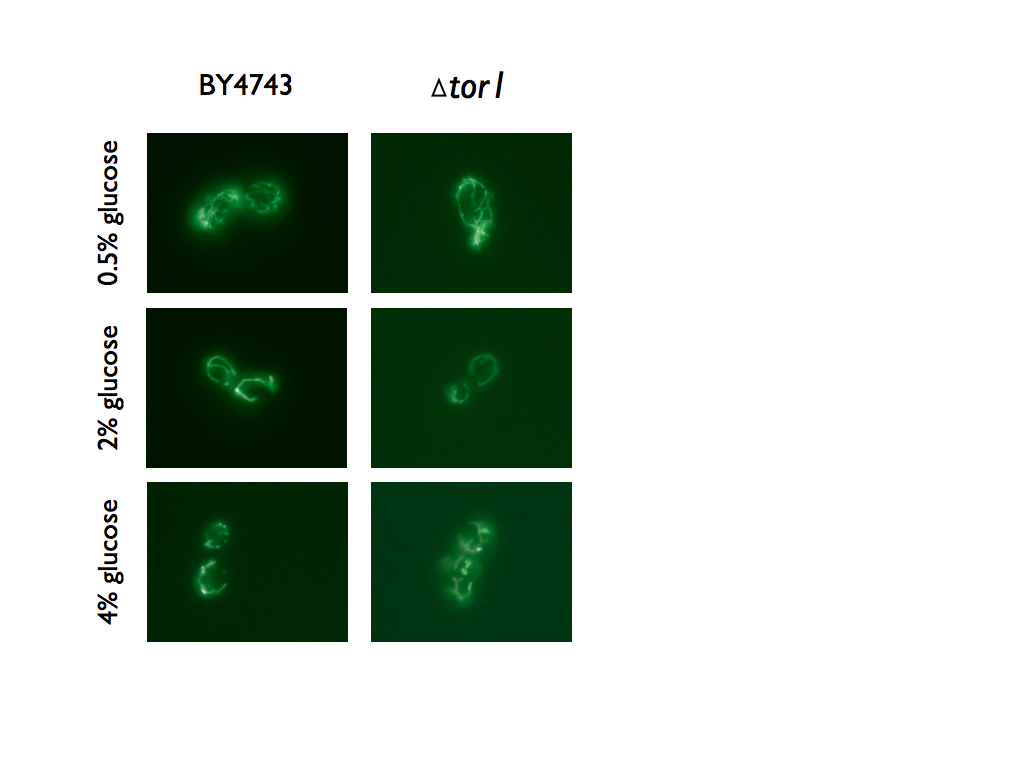
**

**Figure S2. Deletion of TOR1 protects cells against mitochondrial fragmentation.** Wild-type and *Δtor1* mutant cells transformed with an *ACO1*-GFP fusion construct were grown for 72 hours in three different concentrations of glucose (0.5%, 2%, and 4%) and the morphology of mitochondria was observed using a fluorescent microscope. The micrographs shown are representative of the population.


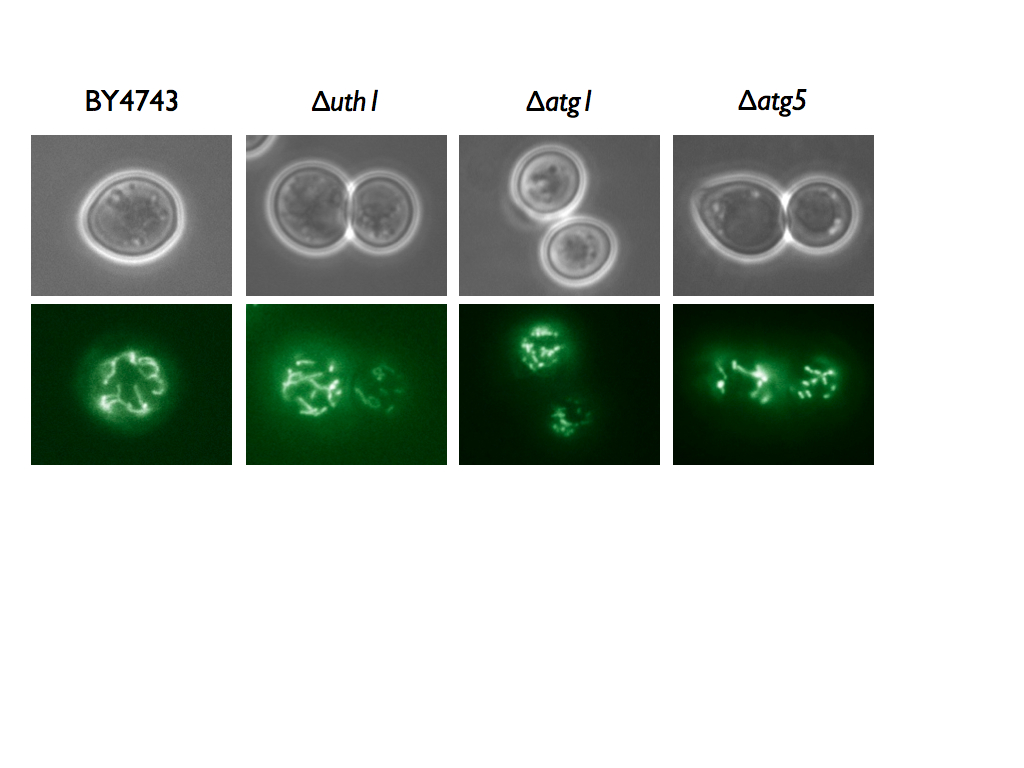


**Figure S3. Rapamycin treatment prevents mitochondrial fragmentation.**

Wild-type and autophagy mutant cells transformed with the *ACO1*-GFP fusion construct were grown for 72 hours in 4% glucose and the morphology of mitochondria was observed using a fluorescent microscope. The micrographs shown are representative of the population.


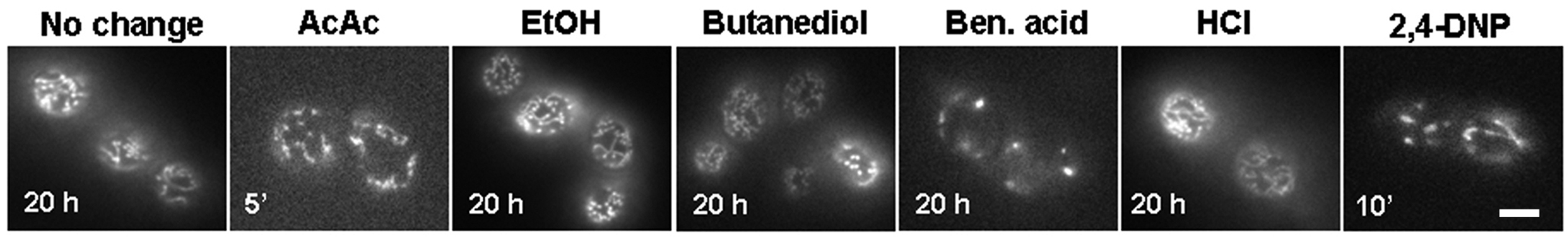


**Figure S4. Acetic acid, benzoic acid and 2,4-DNP triggered mitochondrial fragmentation in *S. cerevisiae*.** The wild-type cells were grown to exponential phase (6 h) in media containing 0.5%, 2% or 4% glucose and then transferred into evaporated 4% conditioned media containing 0.2% (w/v) acetic acid (AcAc), 2% (w/v) ethanol (EtOH), 2% (w/v) butanediol, 2 mM benzoic acid (Ben. acid), HCl pH 3.0 (HCl) and 2.5 mM 2,4-dinitrophenol (2,4-DNP) and mitochondrial morphology was observed at the indicated times. The pH of all media was adjusted to 3.0. Only micrographs of the cells grown in 0.5% glucose are shown here. Scale bar: 4 μm.

**Figure. S5.** **The intracellular pH of cells grown in different concentrations of glucose.** (A) Calibration curve for intracellular pH measurement. The ratio of fluorescent intensity at 405/488 nm for cultures with a range of pH (5.0-8.0) prepared as described in the Experimental Procedures. (B) The ratio of fluorescent intensities at 405/488 nm of the wild-type cells grown for 24 h in medium containing 0.5% (grey line), 2% (grey filled) and 4% (black line) glucose. The median intracellular pH for the 0.5% glucose culture was 7.3, for the 2% glucose culture was 6.55 and for the 4% glucose culture was 6.85.
